# Pancreatic involvement in patients with inborn errors of metabolism

**DOI:** 10.1186/s13023-021-01685-9

**Published:** 2021-01-20

**Authors:** Woo Jin Hwang, Han Hyuk Lim, Yoo-Mi Kim, Mea Young Chang, Hong Ryang Kil, Jae Young Kim, Wung Joo Song, Harvey L. Levy, Sook-Za Kim

**Affiliations:** 1 Department of Pediatrics, College of Medicine, Chungnam National University, Daejeon, Korea; 2 Department of Pediatrics, Gyeongsang National University Hospital, Changwon-si, Gyeongsangnam-do Korea; 3 Korea Genetics Research Center, Cheongju, Korea; 4 Division of Genetics and Genomics, Boston Children’s Hospital, Harvard Medical School, Boston, USA

**Keywords:** Pancreatitis, Diabetes mellitus, Inborn errors of metabolism, Methylmalonic aciduria, Propionic acidemia, Newborn screening, Hyperornithinemia, Fatty acid oxidation disorder

## Abstract

**Background:**

Repeated inflammation of the pancreas can cause pancreatitis or diabetes. It is well recognized that the organic acidemias may be complicated by pancreatitis but less recognized are other metabolic disorders in which pancreatitis can occur. This study shows that long-term follow-up of patients with various metabolic disorders in Korea revealed several with episodes of isolated pancreatitis or diabetes concomitantly with pancreatitis.

**Results and discussion:**

In this study, two patients with methylmalonic aciduria (MMA), two with propionic acidemia (PPA), one with fatty acid oxidation disorder (FAOD), and one with hyperornithinemia, gyrate atrophy, and juvenile onset diabetes mellitus (DM) were clinically followed for up to 10 – 21 years. Two Korean siblings with MMA showed recurrent pancreatitis from the age of 15 and 19, respectively. The frequency of admission due to pancreatitis was up to 11 times. One patient with MMA developed diabetes mellitus at the age of 20. The other patient with MMA developed recurrent pancreatitis at 4 years and diabetes at 8 years of age. One of the patients with PPA presented with diabetic ketoacidosis. The other PPA patient died of cardiac arrest at age 10. The patient with FAOD presented with pancreatitis at 10 years and died at the age of 15 years due to cardiac arrest. A 35-year-old woman with hyperornithinemia/gyrate atrophy was diagnosed with juvenile onset diabetes at the age of 7 years. No pancreatitis occurred during the follow-up period.

**Conclusions:**

We conclude that various metabolic disorders can trigger acute or chronic pancreatitis. Proper and prompt multidisciplinary management of metabolic derangement is crucial for preventing pancreatic damage. Further clinical and investigational studies are required to elucidate the pathogenesis of pancreatitis and diabetes mellitus in patients with inborn errors in metabolism.

## Introduction

Pancreatitis is the inflammation of the pancreas. Recurrent inflammation can damage the pancreas and progress to chronic pancreatitis. Fibrosis may form in the pancreas, resulting in loss of pancreatic function. A poorly functioning pancreas can cause digestive problems, abdominal pain, and diabetes mellitus (DM). The most common causes of acute pancreatitis (AP) in adults are alcohol consumption and presence of gallstones; however, in children, it is associated with a wide variety of potential etiologies, including idiopathic, trauma, drugs (asparaginase, mesalazine, opiates, etc.), infections (mumps, Epstein-Barr virus, Cytomegalovirus), systemic diseases (systemic lupus erythematosus, Henoch-Schonlein purpura, cystic fibrosis), and hereditary (Kazal type 1 gene (SPINK1) increase the susceptibility of chronic pancreatitis, PRSS1 is mutations of cationic trypsinogen gene) and congenital anatomic anomalies [1]. Organic acidemia was first reported in 1994 as yet another cause of pancreatitis and accounts for 7% of the total pediatric cases of AP [2–7]. Although pancreatitis has been described in association with other disorders, even including phenylketonuria (PKU), [8] and in a Korean patient [9] pancreatitis is less well known in other categories of metabolic disorders [10, 11] and in Korean patients.

In cases of metabolic pancreatitis, management of the underlying causes is more important than the standard routine management of pancreatitis. The lack of proper metabolic management can lead to recurrent bouts of AP [11].

Herein, we report a Korean experience with AP and complications in different metabolic disorders. The complications include recurrent pancreatitis and DM. Our report emphasizes the occurrence of pancreatitis in different metabolic disorders among Korean patients and the need for careful metabolic therapy to lower the risk of pancreatitis and also to treat AP when it occurs.

## Patients and methods

Six patients (5 men and 1 woman) with inborn errors of metabolism who had experienced complications involving the pancreas were included in this study.

Two patients with methylmalonic aciduria (MMA), two patients with propionic acidemia (PPA), one patient with fatty acid oxidation disorder (FAOD), and one patient with hyperornithinemia, gyrate atrophy, and juvenile-onset DM were clinically followed up for 10–20 years at the Chungnam National University Hospital and/or Kim SZ Children’s Hospital.

The six patients were evaluated clinically. Laboratory evaluation included serum amylase and lipase tests, renal clearance assay based on urine creatinine level, and pancreatic auto antibody tests.

Serum amylase and lipase levels were measured using colorimetric assays, where a spectrophotometer was used to measure the absorbance of the samples at 420 nm. A tandem mass spectrometer (MS/MS) (Micromass Quattro spectrometer) was used for metabolic screening. The plasma and urine amino acid levels were measured using an ion exchange chromatography amino acid analyzer (Hitachi high-speed amino acid analyzer L-8900). Renal clearances were calculated based on the urine creatinine level. Organic acid content was measured using gas chromatography-mass spectrometry (Agilent). Pancreas autoantibody tests were performed at other laboratories.

Metabolic tests for inborn metabolic disorders were performed using tandem mass spectrometry (metabolic screening), plasma and urine amino acid analysis, and organic acid analysis.

We applied the diagnostic criteria of AP (at least two of the following three features):

1. Acute onset of upper abdominal pain, nausea, vomiting
2. Serum amylase and/or lipase ≥ 3 times the upper limit of the normal local range
3. Imaging findings characteristic of AP

The diagnostic criteria of DM symptoms included polyuria/polydipsia/weight loss, fasting glucose > 126 mg/dL or random glucose > 200 mg/dL, and hemoglobin A1c ≥ 6.5%.

Autoimmune diabetes was determined by the presence of autoimmunity against one or more of the following antibodies: islet cell autoantibodies (ICA), autoantibodies to glutamic acid decarboxylase (GAD), and insulin autoantibodies (IAA).

## Case reports

The pancreatic involvement of patients is summarized in Table 1. Their clinical manifestation and biochemical findings are shown in Table 2.

**Table 1:** Pancreatic manifestation and precipitating factors of hereditary metabolic disorders

	Age (years)	Number of hospitalizations	Precipitating factors	Pancreatic disorders
MMA (Case 1)	15 16 19 20	1 3 2 1	Dehydration, fever, poor oral intake, liver dysfunction	Pancreatitis, Diabetes, Mellitus
MMA (Case 2)	21	1	Respiratory infection, weight loss, bronchial, asthma, vomiting, dehydration	Pancreatitis, Diabetes
PPA (Case 3)	14	1	Diabetes mellitus for 14 years	Diabetes
PPA (Case 4)	4 5 6 8	1 2 1 1	Surgery for intestinal obstruction, swine flu, fever, dehydration, ferritinemia	Pancreatitis, Diabetes
FAO (Case 5)	10	1	Fasting, dehydration	Pancreatitis
Ornithine (Case 6)	33	None	No diet control for ornithine	Diabetes

**Table 2:** Clinical history of patients with hereditary metabolic disorders

	Case 1	Case 2	Case 3	Case 4	Case 5	Case 6
Underlying disease	MMA	MMA	PPA	PPA	FAO	Hyperornithinemia
Age/Sex	20 y/M	15 y/M	14 y/M	10 y/M	15 y/M	35 y/M
Birth history	37 wks, 2.7 kg, C/sec	GA 37 wks, 2.47 kg, C/sec	GA 36 wks, 3.2 kg, C/sec	Full term, 3.7 kg, NSVD	Full term, NSVD	Full term, 3.73 kg, NSVD
Age of diagnosis	Perinatal period	Perinatal period	Perinatal period	Perinatal period	40 m	7 y
NH_3_ (μg/dL)	500	> 1000	400	1200	300	Normal
Management	Special formula (Propimex) Low-protein diet L-carnitine supplement		Low-protein diet Special formula (MPA)		Freq. feeding Raw corn starch	Insulin Low-protein diet

### MMA cases

Case 1 was a 20-year-old man with MMA. He was born via cesarean section because of placenta previa at gestational age of 37 weeks with birth weight of 2.7 kg. Due to positive family history (his brother had MMA), he underwent metabolic investigation. His metabolic evaluation revealed blood ammonia level of 400–500 mg/dL and elevated C3 carnitine level (48.8; reference range, < 4.1). The methylmalonic acid level in urine was 1617 µg/mg Cr, and elevation in plasma glycine (415 µmol/L) and alanine (978 µmol/L) levels were noted. Cobalamin (Cbl) incorporation study of 14C-propionate showed lack of response to Cbl (i.e. mut^0^) and slightly low response to adocobalamin (AdoCbl). Molecular investigations revealed a compound heterozygous c.1105C > T(p.R369C), p.G94Q mutation. He was fed with special formula (Propiomex), regular infant formula, low-protein food, and L-carnitine supplement (100 mg/kg/d). His developmental evaluation at 20 months of age showed a mild delay. Prior to 5 years of age, blood urea nitrogen (BUN) and creatinine were in the normal range (BUN 6.6–12.3 mg/dL /creatinine 0.7–0.8 mg/dL). However, after 5.5 years of age, his renal function slowly deteriorated, showing a BUN level of 16.2–29.2 and creatinine level of 0.81–1.02. He was hospitalized multiple times due to metabolic decompensations (acidosis, dehydration, and hyperammonemia) after trivial infections or skipping of the special formula. At 6 years of age, his eye sight deteriorated progressively and he became legally blind due to optic atrophy by 10 years of age. He received growth hormone treatment for metabolic induction to anabolism and improvement of his short stature. At 15 years of age, he experienced vomiting and abdominal pain due to pancreatitis. His metabolic status became unstable; he was hospitalized more than 7 times with pancreatitis (Table 1). At this time, he began to experience hand tremors due to his metabolic condition. After recurrent pancreatitis at 19 years of age, he showed glucose intolerance. By the age of 20 years, he developed DM, requiring insulin injection. His most recent metabolic crisis was accompanied by severe metabolic acidosis (pH 6.8), liver dysfunction (aspartate aminotransferase (AST) 245 IU/L, alanine aminotransferase (ALT) 316 IU/L), hyperventilation, hemorrhagic gastritis, and left pleural effusion. His pancreatic amylase and lipase levels were > 4000 U/L. He was put on continuous renal replacement therapy for 3 days. During the metabolic crisis, he experienced seizures and hand tremors, although the results of brain magnetic resonance imaging (MRI) and electroencephalogram (EEG) were normal. The abdominal computed tomography (CT) showed necrotizing pancreatitis and follow-up ultrasound after 1 month of pancreatitis showed a pancreatic pseudocyst. His renal function progressively deteriorated, showing a BUN level of 40.1–60 mg/dL, Cr level of 2.04–2.81 mg/dL, and estimated glomerular filtration rate (eGFR) level of 43.7 mL/min/1.73 m^2^. His metabolic parameters were as follows: pH, 7.30–7.31; HCO_3_ level, 11.2–20.6; methylmalonic acid level, 1768–3861 µg/mg Cr; glycine level, 625–1187 µmol/L (normal range: 158–302 µmol/L); alanine level, 508–825 µmol/L (normal range: 185–537 µmol/L). His ferritin levels were 280–581.53 ng/mL (normal range: 50–200 ng/mL). His current weight is 46.1 kg and height is 164 cm. He has recovered completely from pancreatitis, and his glucose levels are being controlled using insulin (Table 2).

Case 2 is the 21-year-old elder brother of Case 1. He was born via cesarean section because of placenta previa at a gestational age of 37 weeks, with a birth weight of 2.47 kg, to a 21-year-old mother who had a history of spontaneous miscarriage. Within a few days after birth, he started vomiting, and showed poor feeding, seizures, and lethargy. He was transferred to a university hospital, where he was found to have blood ammonia level of over 1000 mg/dL and acute metabolic acidosis. At 2 weeks of age, he was diagnosed with MMA by urine organic acid analysis. After peritoneal dialysis, he was in the neonatal intensive care unit (NICU) for 2 weeks for metabolic control. Until 4.5 years of age, multiple hospitalizations for metabolic crises followed, as a special metabolic formula was not available in South Korea at that time. After obtaining a special formula (Propiomex) from overseas, regular baby formula and low-protein food became a part of the ongoing metabolic management and his metabolic status was stabilized. Cbl incorporation study of 14C-propionate showed no response to cobalamin (i.e. mut^0^) and slightly low response to AdoCbl. Similar to Case 1, the molecular study revealed the presence of the 1105C > T(p.R369C), p.G94Q mutation. He began receiving growth hormone treatment at the age of 11 years for metabolic induction to anabolism and improvement of his short stature. His biochemical parameters were as follows: pH, 7.29–7.4; pCO_2_, 38.1; pO_2_, 62.8; HCO_3_ level, 9.6–23.6; base excess level, –1.1; SO_2_ level, 82–93.5%. His blood ammonia level was 84–126 µg/dL, lactic acid level was 2.0 mmol/L (normal range: 0.5–2.2 mmol/L), HCO3 level was 9.6–17.7 mmol/L (normal range: 21–27 mmol/L), and TG level was 252–291 mg/dL (normal range: 34–143 mg/dL). His renal function was poor, with a BUN level of 45.6–74.5, Cr level of 2.8–3.0, and eGFR of 20.7–27.5 mL/min. Between the age of 19 and 21 years, chronic and recurrent pancreatitis was observed, with total amylase level of 176–215 IU/L (normal range: 25–125 IU/L) and lipase level of 100 U/dL (normal range: 10–140 U/dL). Pancreatitis was resolved rapidly via administration of 10% dextrose electrolyte intravenous (IV) fluid, discontinuation of oral feeding, and placement of a nasogastric tube. Biochemical evaluation revealed a C3 carnitine level of 67.6 (normal range: > 4.1), glycine level of 824 mmol/L (normal range: 158–302 mmol/L), alanine level of 703 mmol/L (normal range: 185–537 mmol/L), glutamine level of 429 µmol/L (normal range: 360–740 µmol/L), methylmalonic acid level of 1182–1444 µg/mg Cr, lactic acid level of 1137 µg/mg Cr (normal range: 6–61 µg/mg Cr), and elevation of the 3-hydroxypropionic and methylcitric acid levels. His current weight and height are 61 kg and 165 cm, respectively. He showed developmental delay (walking and speech at the ages of 20 months and 43 months, respectively). He is now independent and has a full-time job at a coffee shop. Recently, he had been hospitalized for headache and decreasing visual acuity. He was diagnosed with essential hypertension and treated with calcium channel blocker.

### PPA cases

Case 3 was a 14-year-old boy. He was born via normal spontaneous vaginal delivery (NSVD) without complication at the gestational age of 36 weeks, with birth weight of 3.2 kg. C3 carnitine level elevation (37, reference: < 4.1) was detected in the expanded newborn screening program. His urine organic acid analysis confirmed PPA. His blood gas and blood ammonia levels were normal. The fibroblast carboxylase assay revealed reduced propionyl-CoA carboxylase (PCC) activity [9 pmol/min/mg protein (reference: > 91 pmol/min/mg)], normal methylcrotonyl-CoA carboxylase (MCC) activity [55 pmol/min/mg protein (reference: > 31 pmol/min/mg)], and normal pyruvate carboxylase (PC) activity [124 pmol/min/mg protein (reference: > 71)]. Propionyl carboxylase assay using biotin-supplemented culture medium showed that he was non-responsive to biotin. He was fed breast milk exclusively and an unrestricted normal regular diet. However, at 23 months of age, he was hospitalized for metabolic decompensation and hyperammonemia following rotavirus gastroenteritis. Special formula for PPA was introduced after that. The patient was not compliant with the PPA formula because of its taste. He was admitted to the hospital 12 times due to metabolic decompensation (i.e. metabolic acidosis and hyperammonemia) at 23 months of age. The unusual clinical presentation was recurrent headache, which was relieved by intravenous glucose/electrolyte infusion. At 13 years, he was diagnosed with Osgood-Schlatter disease. At 14 years of age, ophthalmologic evaluation revealed Avellino corneal dystrophy. He experienced diabetic ketoacidosis, which was associated with weight loss. His blood glucose level was 2018 mg/dL and C-peptide level was 0.87 ng/mL (reference: 0.81–3.86 ng/mL). His autoantibody profile was as follows: islet cell antibody (Ab)-negative, insulin Ab-negative, and anti-GAD Ab level < 0.11 (reference: ≤ 1.00 U/mL). Insulin was administered to the patient, after which he became compliant with the PPA special formula for diet control (Table 2). His long-term metabolic profile revealed glycine level of 261–314 µmol/L (normal range: 158–302 µmol/L), significantly elevated glutamine level of 1169–1545 µmol/L (normal range: 360–740 µmol/L), and small amounts of 3-hydroxypropionic acid, propionyl glycine, and methyl citrate. His amylase level had always been normal at 81–90 U/L (reference: 28–100 U/L) during our long-term follow-up.

Case 4 was a patient with PPA. He was born via NSVD at full-term without complications, and his birth weight was 3.7 kg. C3 carnitine level elevation (63, reference: > 4.1) was detected in the expanded newborn screening program. In the initial clinical evaluation, he was icteric, sleeping without feeding (dry oral mucous membrane), and in a drowsy mental state. His blood ammonia level was 900–1200 mg/dL and urine ketone level was + 3. Amino acid analysis showed elevated levels of glycine (1178), glutamine (1250), and alanine (600). Urine organic acid analysis revealed elevated levels of methylcitric acid, tiglylglycine, 3-hydroxypropionic acid, propionyl glycine, and lactic acid (1.6 mmol/L), confirming PPA. He received peritoneal dialysis, but the dialysis was complicated by peritonitis, intestinal obstruction, and adhesion 68 days after birth. He had recurrent pancreatitis at the age of 4, 5 (twice), 6, and 8 years (Table 1). At 6 years of age, he had swine flu, which acutely compromised his immune system; he thus required ICU care for one year. He had pleural and pericardial effusion, atelectasis in the left lower lobe, and ascites. He also showed inappropriate secretion of the antidiuretic hormone (syndrome of inappropriate antidiuretic hormone secretion, SIADH). Following multiple blood transfusions for anemia, his ferritin level was > 3000 ng/mL (normal: 300 ng/mL). He was administered deferoxamine to lower the ferritin levels. At the age of 8 years, he developed diabetes, which was controlled by insulin. His weight and height remained at 20 kg and 133 cm, respectively, until his sudden death by cardiac arrest at the age of 10. The results of his autoantibody tests for diabetes were all negative. His amylase and lipase levels stayed elevated at 317–558 IU/L (normal range: 25–125 IU/L) and 347–1585 U/dL (10–140 U/dL), respectively.

### Case of fatty acid oxidation disorder

Case 5 was a 15-year-old boy with cerebral palsy. He was born via NSVD at full term. He showed normal growth and development until 40 months of age, which was when he had seizures followed by fever of unknown origin. He underwent a day-long fasting for sonogram, which resulted in acute liver enlargement and elevated liver enzyme levels. The sonogram showed fatty liver and hepatomegaly. Following the fasting, he had cerebral palsy with spasticity. The acylcarnitine profile showed elevated free carnitine levels, no ketones in urine, and generalized aminoaciduria. Urine acylglycine analysis revealed a propionyl glycine level of 3.81 (reference: < 1.93), and urine organic acid analysis revealed hypoketotic hypoglycemia and 3-methylglutaconic aciduria during a metabolic crisis. He experienced periodic pseudo gut paralysis, requiring metronidazole treatment. Muscle biopsy showed normal mitochondria, although the number of mitochondria was low. At 10 years of age, he entered an unresponsive semi-comatose state following poor oral food intake for over 6 days, which was accompanied by marked hepatomegaly, severe dehydration, and pancreatitis. His liver enzymes were elevated as follows: AST, 4428 U/L; ALT, 2532 U/L; however, his bilirubin level was normal (0.2–0.3 mg/dL). At the same time, his renal function was considerably impaired (BUN level, 120 mg/dL (reference: 18 mg/dL); Cr level, 2.6 mg/dL; lactic acid level, 2.9 mmol/L; NH_3_ level, 200–300 mmol/L). Other laboratory findings showed the following: sodium level, 140; chloride level, 107 mmol/L; phenylalanine level, 720 (normal range: 39–76); free carnitine level, 80.2 (reference: ≤ 50); LDH level, 456 U/L (normal range: 100–190 U/L); CPK level, 446 mg/dL (normal range: 21–232 mg/dL); lipase level, 493–13,978 U/dL (normal range: 10–140 U/dL); amylase level, 122.0–808 U/dL (normal range: 25–125 U/dL) (Table 2). Abdominal CT scan showed edematous changes and enlarged pancreas. Pancreatitis was alleviated in 2 weeks by supportive treatment involving the administration of 10% dextrose electrolyte solution. His extra-pancreatic manifestations were pleural effusion, pneumonia, and scoliosis of the T-L spine. At 11 years of age, he had status epilepticus after prolonged inadequate oral food intake because of parental refusal to undergo gastrostomy tube placement. He again experienced liver dysfunction and progressive hepatomegaly. Brain MRI revealed severe brain atrophy with dilatation of ventricles and prominent sulci. After recurrent aspiration pneumonia, pseudo gut paralysis, and liver dysfunction, he died of a cardiac arrest at the age of 15 years.

### Gyrate atrophy with hyperornithinemia

Case 6 was a 35-year-old woman who was confirmed to be blind and diabetic. She was born at full term via NSVD to a 21-year-old mother (G2P1 SAB) with a birth weight of 3.73 kg. She had tunnel vision, myopia, and night blindness at the age of 5 years. At the age of 7, she was diagnosed with juvenile-onset diabetes. She had menarche when she was 16 years old. She was evaluated metabolically at the age of 34 and high ornithine levels were detected in her blood and urine. Hyperornithinemia/gyrate atrophy was confirmed. She was legally blind, but had been working with constricted visual field at a daycare center. She had normal chromosomes (46, XX). The pancreatic autoimmune panel test was negative for the GAD protein and islet cell antibody; however, a high titer of anti-insulin antibodies was observed, which might be due to insulin therapy. Her vital signs, including blood pressure (110/70 mm Hg), were stable. Her weight was 57.1 kg (50th percentile), height was 156 cm (10th percentile), and HC was 54.5 cm (25–50th percentile). She looked relatively healthy and had a round face. Physical examination showed short stature and sparse light and dark hair. Her lower thoracic cage was prominent. Her lungs were clear to auscultation and heart sounds were normal without murmurs. Regarding the extremities, she had short limbs, thick and short fingers, missing knuckles of the 4th fingers, abnormally long 2nd and 4th toes, pitting edema, and mildly short Achilles tendons. She had subcutaneous nodules on both legs, lipodystrophy on the injection sites of the abdomen, and brisk DTR. In addition, her visual field was extremely constricted and her fundi showed abnormal pigmentation, sparing a small proportion of the retina. Ophthalmologic examination revealed isocoric pupils, clear corneas, pale fundi, and thin retinal vessels; she also had limitations of extraocular muscle movements in all directions, with nystagmus. Plasma amino acid analysis showed ornithine levels of 756–900 µmol/L (reference: 106 µmol/L). After the diagnosis of gyrate atrophy of the choroid and retina associated with hyperornithinemia, she was fed a low-protein diet (Table 2). During the follow-up, she did not have pancreatitis (Table 3).

**Table 3:** Laboratory data of hereditary metabolic disorders

Underlying disease	MMA (Case 1)	MMA (Case 2)	PPA (Case 3)	PPA (Case 4)	FAOD (Case 5)	Hyperornithinemia (Case 6)
*Pancreatitis*						
Pancreatitis	+	+	−	+	+	−
Amylase during metabolic crisis (IU/L)	> 4000	176–215	81–90	317–558	122–808	−
Lipase (range)	> 4000	100	−	347–1585	493–13,978	−
Onset of AP	15 y	19 y	−	4 y	10 y	−
No. of episodes	7	1	1	5	1	None
*Diabetes mellitus (DM)*						
DM	+	−	+	+	−	+
DM_onset	20 y	−	14 y	8 y	−	7 y
DM_management	Insulin	−	Insulin	Insulin	−	Insulin

*MMA* methylmalonic academia, *PPA* propionic academia, *FAOD* fatty acid oxidation disorder, *HO* hyperornithinemia

## Discussion

In this report we described pancreatitis in 6 Korean patients with metabolic disorders, 4 with an organic acid disorder, one with a fatty acid oxidation disorder, and one with an aminoacidopathy. Thus, our experience over a period of 10–20 years emphasizes the ubiquity of pancreatitis among the metabolic disorders as well as the need for careful and complicated metabolic therapy when pancreatitis occurs.

A large number of patients with pancreatitis develop glucose intolerance or DM; islet β-cell injury in pancreatitis may explain the occurrence of glucose intolerance or DM. However, α-cell function can be equally important, as selective α-cell destruction prevents the hyperglycemic response associated with pancreatitis [12]. It has been accepted that injury to islet cells in chronic pancreatitis results in insulin deficiency; however, normal or elevated fasting plasma insulin levels in patients with chronic pancreatitis do not support the expected findings [13, 14].

In this report, Case 1 (MMA) had both diabetes and pancreatitis, but Case 3 (PPA) and Case 6 (hyperornithinemia with gyrate atrophy) had diabetes with normal pancreatic enzyme levels. All three patients showed negative results in the pancreatic autoantibody panel. These findings may suggest the presence of chronic adverse alterations in pancreatic cells due to metabolic insults and toxic compounds associated with metabolic disorders.

Not only is acute and chronic accumulation of organic acids toxic to pancreatic cells, but lactic acid, uric acid, and acidosis may also affect the injury to pancreatic tissue. Reports show that glycogen storage disease type I (von Gierke disease) is associated pancreatitis [15–18]. The mechanism underlying the development of pancreatitis has been elucidated, although the most common biochemical changes in the disease include hypoglycemia, lactic acidosis, and high levels of uric acid and triglycerides—any of which may contribute to the onset of pancreatitis. Previous studies have reported the incidence of pancreatitis in organic acidurias; the first ever report discussed pancreatitis in 9 out of 108 patients with branched-chain organic aciduria (BCOA) [2, 7], while pancreatitis was observed in 10 patients with isovaleric acidemia [19, 20], 2 with propionic academia [4, 6], 1 with MMA [6], 2 with maple syrup urine disease (MSUD) [21], 1 with glutaric academia [5], and 2 with 3-hydroxy-3-methylglutaryl-CoA lyase deficiency [22, 23]. Case 5, who had a fatty acid oxidation metabolic disorder, showed elevated transaminase levels and hepatomegaly after prolonged fasting or inadequate diet, which led to pancreatitis. Lack of energy supply could be a precipitating factor of pancreatitis. Treatment involving glucose electrolyte solution alleviated the hepatomegaly and decreased the transaminase level to normal. The liver function profile showed elevation of AST and ALT levels, with normal levels of bilirubin. Case 6 (hyperornithinemia with gyrate atrophy) has DM but, interestingly, pancreatitis was never noted in her medical record or during numerous clinical follow-ups emphasizing the need for diligence in any patient with a metabolic disorder who develops DM. Lipid metabolic disorders such as hyperlipidemia appear to be one of the most common inherited causes of recurrent pancreatitis. This includes hereditary lipoprotein lipase deficiency [24–27], familial hypertriglyceridemia, chylomicronemia (type I & V), hyperlipoproteinemias [28], and apolipoprotein C-II deficiency. In particular, pancreatitis represents frequent and sometimes severe complications of apolipoprotein C-II deficiency; up to 60% patients with apolipoprotein C-II deficiency are affected by episodes of pancreatitis [29–31]. Hyperlipidemia interferes with amylase measurements, leading to false-negative results [20, 21, 26, 27, 32, 33]. This may explain the normal or mildly elevated amylase levels in patients with severe necrotizing pancreatitis.

The observations regarding other amino acid metabolic disorders varied. Postmortem histology of patents with homocystinuria (cystathionine β-synthase deficiency) and pancreatitis has revealed marked fibrous thickening of the intima, and frayed and split muscle and elastic fibers of the media without lipid deposition in most large- or medium-sized arteries [28, 34, 35]; thus, blood circulation to the pancreas may have been compromised. Cystinuria is associated with familial pancreatitis [36]. Duct calculi in patients with cystinuria, chronic renal insufficiency or renal failure may adversely affect the pancreas [37, 38]. Lysinuric protein intolerance leads to inflammatory changes, necrosis, intraductal protein plugs, atrophy, and fibrosis in the pancreas [39, 40]. Furthermore, pancreatitis has been reported in patients with pyruvate kinase deficiency [41] and acute intermittent porphyria [42–46].

## Conclusion

Pancreatitis is not a common complication of the inborn errors of metabolism, and is often overlooked during the treatment and management of patients with metabolic disorder. Yet, acute pancreatitis can accompany abdominal pain, exacerbate vomiting episodes, and cause subsequent rapid metabolic deterioration. Acute or chronic pancreatitis may complicate the condition of patients with inborn errors of metabolism. Pancreatitis must be considered in patients with these disorders who have acute clinical deterioration and vomiting, abdominal pain, encephalopathy or shock, or milder symptoms. Inborn error of metabolism should be considered in children with pancreatitis of unknown origin. While the pathophysiology may vary in different cases, pancreatitis should be monitored not only in cases of metabolic disorders involving hyperlipidemia, but also in cases of amino acid, organic acid, and fatty acid oxidation disorders, as well as in cases of heme and pyruvate metabolic disorders.

Early diagnosis and management of pancreatitis may prevent long-term complications such as type 2 DM. Further studies are required to elucidate the pathogenesis of pancreatitis, as well as of DM associated with hereditary metabolic disorders.

## Data Availability

All data generated or analysed during this study are included in this published article and its supplementary information. The datasets used and/or analysed during the current study are available from the corresponding author on reasonable request.

## Abbreviations

MMA: Methylmalonic aciduria
PPA: Propionic acidemia
FAOD: Fatty acid oxidation disorder
DM: Diabetes mellitus
AP: Acute pancreatitis
MS/MS: Tandem mass spectrometer
ICA: Islet cell autoantibodies
GAD: To glutamic acid decarboxylase
IAA: Insulin autoantibodies
AdoCbl: Adocobalamin
Cbl: Cobalamin
BUN: Blood urea nitrogen
AST: Aspartate aminotransferase
ALT: Alanine aminotransferase
MRI: Magnetic resonance imaging
EEG: Electroencephalogram
CT: Computed tomography
NICU: Neonatal intensive care unit
NSVD: Normal spontaneous vaginal delivery
PCC: Propionyl-CoA carboxylase
MCC: Methylcrotonyl-CoA carboxylase
PC: Pyruvate carboxylase
NSVD: Normal spontaneous vaginal delivery
SIADH: Syndrome of inappropriate antidiuretic hormone
ICU: Intesive care unit
LDH: Latic dehydrogenase
CPK: Creatine phospokinase
GAD: Generalized Anxiety Disorder
HC: Head circumference
DTR: Deep tendon reflex
BCOA: Branched-chain organic aciduria
